# Association of Evening Meal-Timing Chronotype with Lower Calcium Intake After Adjustment for Diet Quality

**DOI:** 10.3390/nu18091376

**Published:** 2026-04-27

**Authors:** Sarang Jeong, Yoon Jung Yang, Sohyun Park

**Affiliations:** 1 Industry-Academy Collaboration Foundation, Dongduk Women’s University, Seoul 02748, Republic of Korea; sarang1031@dongduk.ac.kr; 2 Department of Food and Nutrition, School of Natural and Information Science, Dongduk Women’s University, Seoul 02748, Republic of Korea; yjyang@dongduk.ac.kr; 3 The Korean Institute of Nutrition, Hallym University, Chuncheon 24252, Republic of Korea; 4 Department of Food Science and Nutrition, Hallym University, Chuncheon 24252, Republic of Korea

**Keywords:** circadian rhythms, chronotype, calcium, diet quality index

## Abstract

**Background:** Evening meal-timing chronotypes often exhibit lower calcium intake; however, whether this relationship remains significant after accounting for overall diet quality remains unclear. This study examined the association between meal-timing chronotypes and calcium intake and evaluated whether this association is maintained after adjusting for overall diet quality. **Methods:** This cross-sectional study analyzed 3465 adults aged 30–49 years from the 2016–2018 Korea National Health and Nutrition Examination Survey. Meal-timing chronotypes were identified using dynamic time warping-based K-means clustering of 24-h energy intake distributions. Survey-weighted linear regression assessed the association between meal-timing chronotype and calcium intake and tested their interaction with the Korean Healthy Eating Index (KHEI; excluding dairy) to evaluate the moderating effect of diet quality. Multinomial logistic regression was conducted to estimate odds ratios (ORs) for low calcium intake according to meal-timing chronotypes. Models were adjusted for age, sex, education, occupation, household income, and physical activity. **Results:** After adjusting for sociodemographic and lifestyle factors, the evening meal-timing chronotype was significantly associated with higher odds of low calcium intake (OR = 2.2, *p* < 0.001). A significant interaction between chronotype and KHEI tertiles on calcium intake was observed (*p* < 0.001). Specifically, while calcium intake generally decreased as diet quality declined, individuals with an evening preference consistently showed significantly lower calcium intake across all KHEI tertiles compared to the morning preference group (*β* = −7.9, *p* < 0.001). **Conclusions:** The evening meal-timing chronotype showed a significant association with lower calcium intake, which remained significant even after accounting for overall diet quality. These findings suggest that circadian-related eating patterns, rather than just overall diet quality, play a structural role in determining calcium intake.

## 1. Introduction

Circadian rhythm is an intrinsic biological cycle that recurs approximately every 24 h [1] and regulates a wide range of physiological and behavioral functions, including sleep–wake cycles, hormone secretion, body temperature, and energy metabolism [2]. Based on interindividual differences in circadian preference, individuals are commonly classified as having morning (“larks”) or evening (“owls”) preferences, reflecting variation in the timing of peak activity and sleep–wake patterns [3,4]. This individual variation in circadian phase is referred to as chronotype and is typically categorized into morning, intermediate, and evening preferences [5].

Chronotype can be assessed using various approaches. Objective indicators, such as sleep midpoint or wake time, have traditionally been used as physiological markers [6], while standardized questionnaires, including the Morningness–Eveningness Questionnaire (MEQ) [7] and the Munich Chronotype Questionnaire (MCTQ) [8], are widely used for subjective assessment. More recently, increasing attention has been focused on characterizing chronotypes based not only on sleep timing but also on the timing of food intake and daily energy distribution [9,10]. In this context, the concept of a meal-timing chronotype, defined using 24-h dietary recall data based on the timing of major energy intake, has been proposed as an alternative approach to capture circadian characteristics reflected in eating behaviors [11]. Compared with sleep-based measures, meal-timing chronotypes may more directly reflect circadian-related patterns of nutrient exposure [12].

Previous studies have shown that individuals with an evening meal-timing chronotype tend to exhibit less favorable dietary behaviors, including irregular meal patterns, breakfast skipping, and increased late-night eating [13]. These behaviors are often associated with poorer overall diet quality, characterized by lower adherence to dietary guidelines and higher consumption of ultra-processed foods [14]. Such patterns have been linked to increased risks of obesity, metabolic syndrome, type 2 diabetes, and cardiovascular disease. In addition, several studies have reported that individuals with an evening preference have lower dietary calcium intake than those with a morning preference [15]. Reviews in the chrononutrition literature consistently indicate that delayed eating patterns are associated with a reduced consumption of calcium-rich foods, particularly dairy products [16]. Evidence from Western populations suggests that delayed meal timing—often accompanied by breakfast skipping—may displace dairy intake, a primary source of calcium that is typically consumed in the morning. Calcium intake is generally considered an indicator of overall diet quality, as calcium-rich foods such as dairy products and certain vegetables are more commonly consumed within structured and regular meal patterns [17]. Therefore, the lower calcium intake observed in individuals with an evening meal-timing chronotype has often been interpreted as part of an overall decline in diet quality associated with unfavorable eating behaviors.

However, it remains unclear whether reduced calcium intake in individuals with an evening meal-timing chronotype reflects overall poorer diet quality or specific temporal patterns of food intake. Because calcium-rich foods are often consumed at particular times of the day, intake may be sensitive to the temporal organization of meals. Nevertheless, research disentangling the relationships among meal-timing chronotype, diet quality, and calcium intake remains limited.

Using data from the 2016–2018 Korea National Health and Nutrition Examination Survey, this study first examined the association between evening meal-timing chronotype and calcium intake. We then assessed the relationship between meal-timing chronotype and overall diet quality. Finally, we evaluated whether the association between evening meal-timing chronotype and lower calcium intake remains significant after adjusting for overall diet quality, as measured by the Korean Healthy Eating Index (KHEI) [18]. In this framework, KHEI was utilized as a key covariate to determine if the observed differences in calcium intake were specifically linked to meal-timing patterns rather than general diet quality. To minimize confounding related to heterogeneous daily schedules, the analysis focused on adults aged 30–49 years—an age group with the highest labor force participation [19]—given that meal-timing chronotypes were derived from the clustering of 24-h energy intake patterns.

## 2. Materials and Methods

### 2.1. Study Data and Participants

This study used data from the 7th KNHANES (2016–2018). Adults aged 30–49 years, representing the demographic group with the highest labor force participation (82.3% for ages 30–39 and 80.6% for ages 40–49, based on the Economically Active Population Survey), were included as the study population. Of the 24,269 individuals surveyed over the 3-year period, 6659 were aged 30–49 years.

Participants who completed all three survey components—health interview, health examination, and nutrition survey—were included, and the corresponding complex sampling weights for each survey year were applied. To minimize the influence of implausible energy reporting, participants with a total daily energy intake within ±3 standard deviations of the mean were retained. To ensure the accurate classification of meal-timing chronotypes, shift workers and individuals with illogical or missing chronological meal-time entries were excluded. Only participants with a regular eating pattern (breakfast–lunch–dinner) were included to maintain homogeneity in circadian rhythm patterns. After applying these criteria, a final sample of 3465 adults aged 30–49 years was included in the analysis.

This study used anonymized secondary data from the Korea Disease Control and Prevention Agency (KDCA). In accordance with the Bioethics and Safety Act, the 2016–2017 KNHANES was exempt from institutional review board (IRB) approval, whereas the 2018 survey was approved by the KDCA IRB (No. 2018-01-03-P-A). This analysis was also approved by the Hallym University IRB (HIRB-2024-062). All data collection procedures and portion size estimations followed official KNHANES guidelines [20].

### 2.2. Sociodemographic and Health-Related Characteristics

Sociodemographic and health-related data were obtained from KNHANES, including age, sex, education level, household income, occupation, alcohol consumption, smoking status, physical activity, and menopausal status. Age was recorded in years. Education level was categorized as elementary school or less, middle school, high school, and college or higher. Household income was stratified into quartiles (low, lower middle, upper middle, and high). Occupation was classified into four categories: professional/office workers (managers, professionals, and clerical workers); manual workers (agricultural and fishery workers, craft and machine operators, and laborers); sales and service workers; and unemployed (including homemakers and students). High-risk alcohol consumption was defined as consuming ≥7 drinks per occasion at least twice per week for men and ≥5 drinks per occasion at least twice per week for women. Smoking status was classified as “current or former smoker” if the individual had smoked at least 100 cigarettes (five packs) in their lifetime; otherwise, participants were classified as “never smokers.” Physical activity was defined according to World Health Organization recommendations: ≥150 min per week of moderate-intensity activity, ≥75 min per week of vigorous-intensity activity, or an equivalent combination. For women, menopausal status included natural and surgical menopause.

### 2.3. Assessment of Calcium Intake Tertiles

Daily calcium intake was estimated using 24-h dietary recall data and categorized into tertiles based on the weighted distribution of the study population. The first tertile (T1) included participants with a calcium intake of 8.9–377.3 mg/day (weighted mean: 265.1 mg/day), the second tertile (T2) included 377.3–591.3 mg/day (weighted mean: 448.2 mg/day), and the third tertile (T3) included 591.4–5031.7 mg/day (weighted mean: 874.5 mg/day). These tertiles were used to examine the distribution of calcium intake across meal-timing chronotypes and to assess the associations between calcium intake levels and chronotype categories.

### 2.4. Dietary Assessment and the KHEI

Dietary intake was assessed using 24-h dietary recall (24-h recall) data from KNHANES. The 24-h recall provided detailed information on food types, portion sizes, and timing of intake, enabling the estimation of individual daily energy and nutrient intakes. Reported food volumes were converted into weight, and mixed dishes were disaggregated into individual ingredients. Nutrient intakes were calculated using the Korean Food Composition Table from the National Institute of Agricultural Sciences.

To assess whether the association between meal-timing chronotype and calcium intake remains significant after accounting for overall dietary patterns, diet quality was evaluated using the KHEI, as provided by KNHANES. The KHEI comprises 14 components across three domains: adequacy (breakfast consumption; mixed grain intake; total fruit intake; fresh fruit intake; total vegetable intake; non-pickled vegetable intake; dairy intake; and meat, fish, egg, and legume intake), moderation (saturated fat energy ratio, sodium intake, and energy from sugars and beverages), and balance (energy ratios from carbohydrates and fat, and overall energy adequacy).

In this study, the dairy component within the adequacy domain was excluded to ensure the independence of the primary outcome (calcium intake) from overall diet quality and to minimize potential overadjustment and multicollinearity, as dairy products are a primary source of dietary calcium. By removing this overlapping component, we aimed to better isolate the association between meal-timing chronotype and calcium intake while accounting for the influence of specific calcium-rich food groups. Accordingly, the modified KHEI score ranged from 0 to 90 and served as a key covariate representing overall diet quality, focusing on dietary diversity and moderation without confounding the primary exposure. To assess the robustness of this approach, a sensitivity analysis using the original 100-point KHEI (including the dairy component) was performed. The results were consistent (Supplementary Table S1), indicating that the observed associations were not driven by this methodological modification.

### 2.5. Classification of Meal-Timing Chronotype Using Dynamic Time Warping

Meal-timing chronotypes were classified based on hourly energy intake (hr1–hr24) derived from 24-h dietary recall data, which reflected dietary intake on the day preceding the interview. According to the KNHANES protocol, the dietary survey ensures that data are collected across all days of the week, thereby capturing a representative combination of weekdays and weekends [21].

For each participant, the proportion of energy consumed at each hour relative to total daily energy intake was calculated. Individual 24-h energy intake profiles were then represented as 24-dimensional vectors and used as input for clustering analysis.

The optimal number of clusters (*K*) was determined using the elbow method by examining the within-cluster sum of squared errors (SSE) [22]. As shown in Supplementary Figure S1, SSE declined sharply from *K* = 1 to *K* = 3, followed by an inflection point at *K* = 3, indicating an optimal and parsimonious solution. This selection was further supported by the silhouette coefficient [23], confirming adequate separation between clusters. Subsequently, *K*-means clustering with dynamic time warping (DTW) distance [24,25]—an alignment-based metric suitable for time-series data—was implemented to group participants based on similarities in their 24-h energy intake patterns. Analyses were conducted using Python 3.13 (Python Software Foundation) with the tslearn library, ensuring the reproducibility and proper alignment of temporally shifted eating patterns. This approach identified three distinct and stable clusters: morning meal-timing chronotype (morning preference), intermediate meal-timing chronotype (intermediate preference), and evening meal-timing chronotype (evening preference).

### 2.6. Statistical Analysis

All statistical analyses were performed using SAS version 9.4 (SAS Institute Inc., Cary, NC, USA). The complex sampling design of KNHANES was accounted for by incorporating strata (kstrata), clusters, and sample weights in all analysis. In this study, meal-timing chronotype was defined as the primary exposure and calcium intake (categorized into tertiles: T1, T2, and T3) as the primary outcome. Categorical variables, including sex, education level, household income, and occupation, were compared across meal-timing chronotype groups using PROC SURVEYFREQ with the Rao–Scott chi-square test and are presented as frequencies and percentages. Continuous variables, such as age, total energy intake, and KHEI score, were analyzed using PROC SURVEYREG and are reported as means ± standard errors. All continuous variables, except age, were adjusted for age, sex, education level, occupation, household income, and physical activity.

Multinomial logistic regression (PROC SURVEYLOGISTIC) was used to estimate odds ratios (ORs) for low calcium intake according to meal-timing chronotypes. To determine whether the association between meal-timing chronotype and low calcium intake remained significant after accounting for overall dietary patterns, the modified KHEI score was included as a primary covariate in the multinomial logistic regression models. Additionally, survey-weighted linear regression models incorporating an interaction term were used to evaluate whether the association between meal-timing chronotype and calcium intake remains consistent across all KHEI tertiles, thereby assessing whether diet quality moderates this relationship. Statistical significance was defined as a two-sided *p*-value < 0.05.

## 3. Results

### 3.1. Average 24-h Energy Intake Ratios by Meal-Timing Chronotype Identified Using DTW Clustering

**Figure 1:**
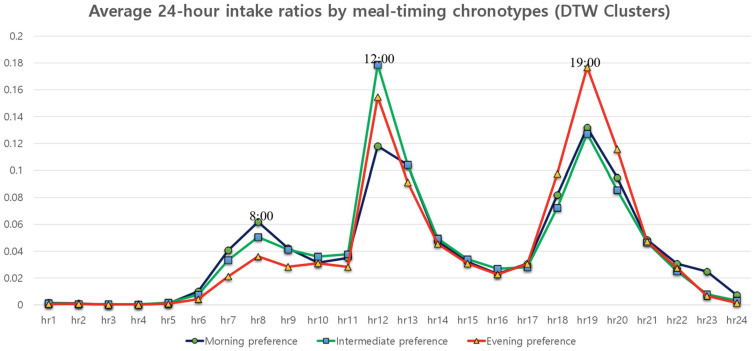
Average 24-h energy intake ratios among study participants aged 30–49 years by meal-timing chronotype identified using dynamic time warping (DTW) clustering. Chronotypes were derived using K-means clustering with DTW distance based on 24-h energy intake distributions. Morning preference: peak intake in the morning (≈08:00), 14.5% of daily energy intake from 7–9 h. Intermediate preference: peak intake at midday (≈12:00), 28.3% of daily energy intake from 12–13 h. Evening preference: peak intake in the evening (≈19:00), 39.0% of daily energy intake from 18–20 h.

The 24-h intake ratio profiles differed markedly across meal-timing chronotypes (Figure 1). During the morning period (07:00–09:00), the morning preference exhibited the highest intake ratio, peaking at approximately 08:00 and accounting for 14.5% of total daily energy intake. During midday (12:00–13:00), the intermediate preference showed the highest intake ratio, with a peak around 12:00 contributing 28.3% of daily energy intake. In contrast, during the evening period (18:00–20:00), the evening chronotype demonstrated the highest intake ratio, peaking at approximately 19:00 and accounting for 39.0% of total daily energy intake. Across all chronotypes, energy intake during overnight hours was minimal.

### 3.2. General Characteristics of Participants by Meal-Timing Chronotypes

Mean age differed significantly across groups, with the lowest mean age observed in the evening preference (*p* < 0.001). Occupational distribution also varied significantly. Non-manual and service or sales workers constituted the majority in the morning preference type, whereas the proportion of manual workers was lowest compared with the intermediate and evening types (*p* = 0.036). The evening preference showed the highest prevalence of high-risk alcohol consumption and smoking (both *p* < 0.001). Conversely, sex, education level, household income, physical activity, and menopausal status did not differ significantly across chronotype groups (Table 1).

### 3.3. Distribution of Calcium Intake Tertiles by Meal-Timing Chronotypes

Table 2 shows significant differences in calcium intake tertiles across meal-timing chronotypes (*p* < 0.001). The morning preference had the highest proportion of participants in the highest calcium intake tertile (37.4%), whereas the evening preference showed the highest proportion in the lowest tertile (39.2%). The intermediate preference displayed an intermediate distribution across tertiles. Overall, calcium intake tended to shift toward lower tertiles from morning to evening preference.

### 3.4. Nutrient Intake and KHEI Scores by Meal-Timing Chronotype Among Participants with Lowest Calcium-Intake Tertile

Table 3 demonstrates nutrient intake and KHEI scores according to meal-timing chronotypes. After adjustment for covariates, total KHEI scores differed significantly across chronotypes (*p* < 0.001), with the lowest score observed in the evening chronotype (53.9 ± 0.5), followed by the intermediate (57.6 ± 0.4) and morning preference (60.6 ± 0.3).

For adequacy components, scores for breakfast consumption, whole grains, fruits (total and fresh), and vegetables were significantly lower in the evening preference compared with the morning preference (all *p* < 0.001). Scores for vegetable intake excluding pickled items and protein sources were also lower in the evening group (*p* = 0.001 and *p* = 0.005, respectively). Accordingly, total adequacy scores decreased progressively from morning to evening preferences (*p* < 0.001).

For moderation components, saturated fat scores differed significantly across groups (*p* < 0.001), whereas sodium and sugar scores did not. However, the total moderation score was lower in the evening preference than in the morning preference (*p* = 0.037). For balance components, scores for carbohydrate and fat energy ratios, energy adequacy, and total balance were all significantly lower in the evening meal-timing chronotype (all *p* < 0.001). Regarding nutrient intake, the evening meal-timing chronotype had significantly lower intakes of calcium, potassium, and dietary fiber, as well as a lower percentage of energy from carbohydrates and total sugar (all *p* < 0.001). In contrast, participants with evening preference had higher total energy intake and higher percentages of energy from total and saturated fats compared with the intermediate preference (all *p* < 0.01). Protein intake did not differ significantly across groups (*p* = 0.916).

### 3.5. Moderating Effect of Diet Quality (KHEI) on the Association Between Meal-Timing Chronotype and Calcium Intake

Table 4 shows a significant interaction between KHEI tertiles and meal-timing chronotype on calcium intake (*p* for interaction < 0.001). In the morning preference, compared with the highest calcium intake tertile (T3), calcium intake was significantly lower in the lowest KHEI tertile (T1; *β* = −41.9, *p* = 0.041), whereas no significant difference was observed for T2. In the intermediate preference, calcium intake decreased progressively across lower KHEI tertiles, with significant reductions observed for T2 (*β* = −56.9, *p* = 0.007) and T1 (*β* = −86.7, *p* < 0.001) relative to the reference group (morning preference within the highest KHEI tertile [T3]). In the evening preference, calcium intake was consistently lower across all KHEI tertiles, with the largest reduction observed in T1 (*β* = −90.0, *p* < 0.001), followed by T2 (*β* = −75.5, *p* = 0.001) and T3 (*β* = −69.2, *p* = 0.002), compared with the same reference group.

## 4. Discussion

This study examined the association between meal-timing chronotype and calcium intake using data from the 2016–2018 KNHANES. The conceptual framework was designed to determine whether an evening meal-timing preference is associated with lower calcium intake after accounting for overall diet quality as measured by KHEI. Our findings suggest that an evening meal-timing preference remained significantly associated with higher odds of low calcium intake (lowest tertile, T1), even after accounting for comprehensive dietary and lifestyle factors.

Notably, compared with individuals with a morning preference in the highest KHEI tertile (T3), those with an evening preference exhibited significantly lower calcium intake across all KHEI tertiles (T1–T3) (Table 4). This indicates that the association between evening preference and lower calcium intake remained significant after statistical adjustment for overall diet quality, suggesting that differences in dietary patterns alone may not fully account for this observed relationship.

Supporting this finding, multinomial logistic regression analysis showed that low calcium intake was significantly associated with an evening preference (Supplementary Table S2), and this association remained robust after adjustment for age, sex, income, and physical activity. Although the primary models were adjusted for diet quality, these additional analyses highlight a structural association between meal-timing chronotype and calcium intake.

Consistent with previous studies [15], individuals with an evening preference in the present study consumed significantly less calcium than those with a morning preference (Table 3). However, beyond replicating this established finding, the present study indicates that calcium intake is associated with meal-timing chronotype even after accounting for KHEI tertiles. This suggests that the lower calcium intake observed in individuals with an evening preference may not be fully explained by poorer overall diet quality and remained evident even after accounting for overall dietary patterns.

These findings may be partly explained by chronotype-specific dietary behaviors. Previous studies have reported that individuals with an evening preference are more likely to skip breakfast [26], consume fewer dairy products and other calcium-rich foods typically eaten in the morning [27], and engage in late-night eating and higher consumption of processed foods [28]. Such behaviors may be linked to restricted opportunities for calcium intake beyond the influence of overall diet quality and are associated with lower diet quality scores.

Overall, this study extends the existing literature by providing evidence of an association between an evening meal-timing preference and lower calcium intake. These findings suggest that lower calcium intake among individuals with an evening preference is not solely attributable to poorer overall diet quality but instead reflects a structurally distinct meal-timing pattern associated with chronotype that is not fully captured by conventional diet quality indices.

As calcium-rich foods are often tied to specific meals, delayed eating patterns may reduce opportunities for calcium intake despite comparable overall diet quality [17]. From a nutritional epidemiology perspective, these findings underscore the importance of considering temporal eating patterns in nutrient assessments. Although causality cannot be determined, the observed association identifies individuals with an evening preference as a high-risk subgroup for persistently low calcium intake, highlighting the need for tailored dietary assessment and intervention strategies.

Several limitations should be acknowledged. First, reliance on a single 24-h dietary recall may introduce within-person measurement error and may not reflect usual intake. Although the simultaneous use of a single recall to classify chronotype and nutrient intake may raise concerns regarding methodological circularity, our findings are consistent with previous studies linking evening-oriented eating patterns to poorer diet quality [29]. Moreover, the large sample size likely mitigates the impact of day-to-day variation at the population level. Additionally, the cross-sectional study design precludes causal inference. The absence of biochemical measures, such as serum vitamin D, parathyroid hormone, and calcium, limits the ability to elucidate underlying biological mechanisms. Future studies incorporating longitudinal dietary assessments and relevant biochemical measurements are needed to determine whether meal-timing chronotype-related differences in calcium intake translate into meaningful changes in calcium metabolism. Collectively, meal-timing chronotype is an important contextual factor in understanding calcium intake patterns and should be considered in the development of individualized dietary recommendations.

This study indicates that an evening preference is associated with lower calcium intake, and this association remained significant after accounting for overall diet quality. This finding suggests that the nutritional vulnerability observed in individuals with an evening preference is not solely attributable to diet quality and may involve biological factors such as circadian misalignment. Previous studies have indicated that circadian misalignment and reduced daytime light exposure can influence calcium homeostasis [30,31]. However, as these physiological parameters were not directly measured in this study, their role remains speculative. Therefore, meal-timing chronotype should be considered a distinct factor in developing tailored nutritional strategies to ensure adequate calcium intake. Future interventions could incorporate chronotype-specific approaches, such as optimizing the timing of calcium-rich food intake or using digital health tools to deliver personalized dietary prompts. Such strategies may effectively address the unique nutritional vulnerabilities of individuals with an evening meal-timing preference by correcting micronutrient imbalances associated with circadian-related eating behaviors.

## Figures and Tables

**Table 1:** General characteristics of study participants aged 30–49 years according to meal-timing chronotype.

		Total (*n* = 3465)			*p*
		Morning Preference (*n* = 1410)	Intermediate Preference (*n* = 1173)	Evening Preference (*n* = 882)	
Age (year)		40.3 ± 0.2 ^b^	40.1 ± 0.2 ^b^	39.3 ± 0.2 ^a^	0.002
Sex	Male/Female	646 (53.8)/ 764 (46.2)	566 (56.2)/ 607 (43.8)	468 (59.5)/ 414 (40.5)	0.050
Education level	Elementary school or less	15 (1.2)	5 (0.3)	6 (0.6)	0.453
	Middle school	47 (3.4)	32 (3.0)	27 (3.5)	
	High school	434 (30.8)	371(30.9)	269 (30.9)	
	College or higher	914 (64.7)	765 (65.8)	580 (65.0)	
Household income	Lowest	58 (4.0)	62 (5.5)	42 (5.1)	0.182
	Upper middle	323 (24.5)	229 (19.9)	195 (21.7)	
	Lower middle	520 (36.2)	409 (35.7)	292 (34.7)	
	Highest	509 (35.4)	473 (39.0)	352 (38.6)	
Occupation	Non-manual workers	326 (24.7)	234 (22.3)	161 (19.3)	0.036
	Manual workers	761 (52.7)	682 (57.3)	501 (56.9)	
	Service and sales workers	198 (13.3)	173 (13.7)	138 (15.1)	
	Unemployed (homemakers, students, etc.)	123 (9.4)	80 (6.8)	80 (8.7)	
Drinking status ^(1)^		183 (14.6)	160 (14.1)	210 (24.7)	<0.001
Smoking status		276 (22.6)	257 (24.1)	254 (32.6)	<0.001
Physical activity ^(2)^		669 (48.4)	542 (48.8)	405 (47.2)	0.822
Menopausal status ^(3)^ (women only, *n* = 1773)		37 (5.2)	31 (5.2)	23 (5.1)	0.997

Data are presented as mean ± standard deviation or weighted frequency (%). The *p*-value for age was obtained using survey-weighted linear regression, while other *p*-values were derived from the Rao–Scott chi-square test. Means with different superscript letters (^a, b^) within a row indicate significant differences at *p* < 0.05 based on Tukey’s post hoc test. ^(1)^ Drinking status was defined as high-risk drinking (men ≥ 7 drinks, women ≥ 5 drinks per occasion, ≥2 times/week). ^(2)^ Physical activity was defined based on compliance with WHO guidelines (≥150 min/week of moderate-intensity activity, ≥75 min/week of vigorous-intensity activity, or an equivalent combination). This composite measure includes general, leisure-time, and transportation-related (walking/cycling) activities to represent total physical activity. ^(3)^ Menopausal status includes natural and surgical menopause.

**Table 2:** Distribution of calcium intake tertiles by meal-timing chronotype among participants aged 30–49 years.

	Total (*n* = 3465)			
Calcium Intake Tertiles	Morning Preference (*n* = 1410)	Intermediate Preference (*n* = 1173)	Evening Preference (*n* = 882)	*p*
Tertile 1	379 (26.5)	423 (35.0)	353 (39.2)	<0.001
Tertile 2	506 (36.1)	394 (34.7)	255 (28.6)	
Tertile 3	525 (37.4)	356 (30.4)	274 (32.2)	

Data are presented as weighted frequency (%). *p*-values were obtained using the Rao–Scott chi-square test. Calcium intake tertiles were defined based on weighted distributions: T1 (8.9–377.3 mg/day; mean 265.1 mg/day), T2 (377.3–591.3 mg/day; mean: 448.2 mg/day), and T3 (591.4–5031.7 mg/day; mean: 874.5 mg/day).

**Table 3:** Comparison of nutrient intake and Korean Healthy Eating Index scores by meal-timing chronotype among participants.

		Total (*n* = 3465)			
		Morning Preference (*n* = 1410)	Intermediate Preference (*n* = 1173)	Evening Preference (*n* = 882)	*p*
**Nutrition Intake**					
Calcium (mg)		561.0 ± 8.9 ^b^	544.7 ± 10.0 ^b^	488.9 ± 9.4 ^a^	<0.001
Total energy intake (kcal)		2161.8 ± 22.8 ^b^	2006.1 ± 27.5 ^a^	2171.8 ± 33.3 ^b^	<0.001
Protein (%E)		14.8 ± 0.1	14.8 ± 0.1	14.9 ± 0.2	0.916
Carbohydrates (%E)		59.9 ± 0.4 ^b^	60.6 ± 0.4 ^b^	53.5 ± 0.6 ^a^	<0.001
Total sugar (%E)		12.9 ± 0.2 ^b^	12.7 ± 0.2 ^b^	11.2 ± 0.2 ^a^	<0.001
Fat (%E)		20.9 ± 0.2 ^a^	21.2 ± 0.3 ^b^	22.5 ± 0.4 ^b^	0.004
Saturated Fat (%E)		6.8 ± 0.1 ^a^	6.9 ± 0.1 ^ab^	7.4 ± 0.2 ^b^	0.005
Potassium (mg)		3022.5 ± 37.7 ^b^	2839.6 ± 45.2 ^a^	2779.7 ± 46.1 ^a^	<0.001
Potassium density/1000 kcal (mg)		1442.3 ± 14.4 ^b^	1445.5 ± 15.9 ^b^	1353.3 ± 16.2 ^a^	<0.001
Sodium (mg)		3769.4 ± 62.5	3666.27 ± 70.2	3677.6 ± 89.6	0.484
Sodium density/1000 kcal (mg)		1769.2 ± 24.4	1856.6 ± 30.2	1765.6 ± 39.8	0.045
Fiber (g)		25.8 ± 0.4 ^c^	24.2 ± 0.5 ^b^	21.5 ± 0.4 ^a^	<0.001
Fiber density/1000 kcal (g)		12.3 ± 0.2 ^b^	12.3 ± 0.2 ^b^	10.7 ± 0.2 ^a^	<0.001
**HEI** ^(1)^					
Total HEI		60.6 ± 0.3 ^c^	57.6 ± 0.4 ^b^	53.9 ± 0.5 ^a^	<0.001
Adequacy items	Breakfast	7.0 ± 0.1 ^c^	6.0 ± 0.1 ^b^	5.3 ± 0.2 ^a^	<0.001
	Whole grain	1.9 ± 0.1 ^c^	1.6 ± 0.1 ^b^	1.3 ± 0.1 ^a^	<0.001
	Fruits (any type)	2.0 ± 0.1 ^b^	1.9 ± 0.1 ^b^	1.7 ± 0.1 ^a^	<0.001
	Fruits (fresh only)	2.3 ± 0.1 ^b^	2.1 ± 0.2 ^b^	1.9 ± 0.1 ^a^	<0.001
	Vegetables (any type)	3.6 ± 0.0 ^b^	3.5 ± 0.1 ^b^	3.3 ± 0.1 ^a^	<0.001
	Vegetables (excluding pickled)	3.3 ± 0.0 ^b^	3.1 ± 0.1 ^a^	3.1 ± 0.1 ^a^	0.001
	Protein sources	7.8 ± 0.1 ^b^	7.4 ± 0.1 ^a^	7.5 ± 0.1 ^ab^	0.005
	Total score	27.8 ± 0.2 ^c^	25.5 ± 0.3 ^b^	24.0 ± 0.3 ^a^	<0.001
Moderation items	Saturated fat (%E)	7.0 ± 0.1 ^b^	6.9 ± 0.2 ^b^	6.1 ± 0.2 ^a^	<0.001
	Sodium	6.3 ± 0.1	6.4 ± 0.1	6.5 ± 0.1	0.436
	Sugar (%E)	9.1 ± 0.1	8.9 ± 0.1	9.1 ± 0.1	0.059
	Total score	22.4 ± 0.2 ^b^	22.1 ± 0.2 ^a^	21.6 ± 0.3 ^a^	0.037
Balance of energy items	Carbohydrates (%E)	3.1 ± 0.1 ^b^	3.0 ± 0.1 ^b^	2.5 ± 0.1 ^a^	<0.001
	Fat (%E)	3.9 ± 0.1 ^b^	3.8 ± 0.1 ^b^	3.2 ± 0.1 ^a^	<0.001
	Energy adequacy	3.4 ± 0.1 ^b^	3.2 ± 0.2 ^b^	2.6 ± 0.1 ^a^	<0.001
	Total score	10.4 ± 0.1 ^b^	10.0 ± 0.2 ^b^	8.3 ± 0.1 ^a^	<0.001

Data are presented as mean ± standard error. *p*-values were obtained from survey-weighted linear regression, adjusted for age, sex, education level, occupation, household income, and physical activity. Calcium intake was additionally adjusted for total energy intake and the KHEI. Means with different superscript letters (^a, b, c^) within a row indicate significant differences at *p* < 0.05 based on Tukey’s post hoc test. ^(1)^ KHEI: Korean Healthy Eating Index. In this study, the total KHEI score was calculated out of a maximum of 90 points by summing 13 components: Adequacy (7 items, 45 points): breakfast (10), mixed grain intake (5), total fruit intake (5), fresh fruit intake (5), total vegetable intake (5), vegetable intake excluding kimchi and pickles (5), meat/fish/eggs/legumes intake (10), and milk and dairy products (excluded). Moderation (3 items, 30 points): Saturated fatty acid energy ratio (10), sodium intake (10), and sugar/beverage energy ratio (10); and balance (3 items, 15 points)—carbohydrate energy ratio (5) fat energy ratio (5), and energy adequacy (5).

**Table 4:** Interaction between Korean Healthy Eating Index tertiles and meal-timing chronotypes on calcium intake among participants.

Meal-Timing Chronotype	KHEI Tertile	Estimate (*β*, Calcium Intake (mg))	Standard Error	*p*
Morning preference	High (T3, reference)	Reference	Reference	Reference
	Middle (T2)	13.1	20.8	0.528
	Low (T1)	−41.9	20.5	0.041
Intermediate preference	T3	−18.7	24.4	0.445
	T2	−56.9	21.1	0.007
	T1	−86.7	22.0	<0.001
Evening preference	T3	−69.2	21.7	0.002
	T2	−75.5	22.7	0.001
	T1	−90.0	20.4	<0.001

*β* coefficients and *p*-values were obtained from survey-weighted linear regression models adjusted for age, sex, education level, occupation, household income, and physical activity. *p* for interaction < 0.001.

## Data Availability

The data used in this study were obtained from the Korea National Health and Nutrition Examination Survey. These data are publicly available through the KDCA website (https:/knhanes.kdca.go.kr/knhanes/main.do (accessed on 13 January 2023)) upon registration and approval. All analyses were conducted following the data-use guidelines of the KDCA.

## Supplementary Materials

The following supporting information can be downloaded at https://www.mdpi.com/article/10.3390/nu18091376/s1, Figure S1: Determination of optimal K using the Elbow method. Table S1: Comparison of nutrient intake and Korean Healthy Eating Index scores by meal-timing chronotype among participants. Table S2: Odds ratios (ORs) and 95% confidence intervals (CIs) for low calcium intake (T1) according to meal-timing chronotype among participants aged 30–49 years.
