# Supplementary material for: Association of Evening Meal-Timing Chronotype with Lower Calcium Intake After Adjustment for Diet Quality
Source: Nutrients. 2026 Apr 27;18(9):1376. doi: 10.3390/nu18091376 (PMC13165129; doi:10.3390/nu18091376)
Supplement: Supplementary file 1 [file nutrients-18-01376-s001.zip › nutrients-4226211-supplementary.pdf]

Supplementary Figure S1. Determination of optimal *K* using the Elbow method.

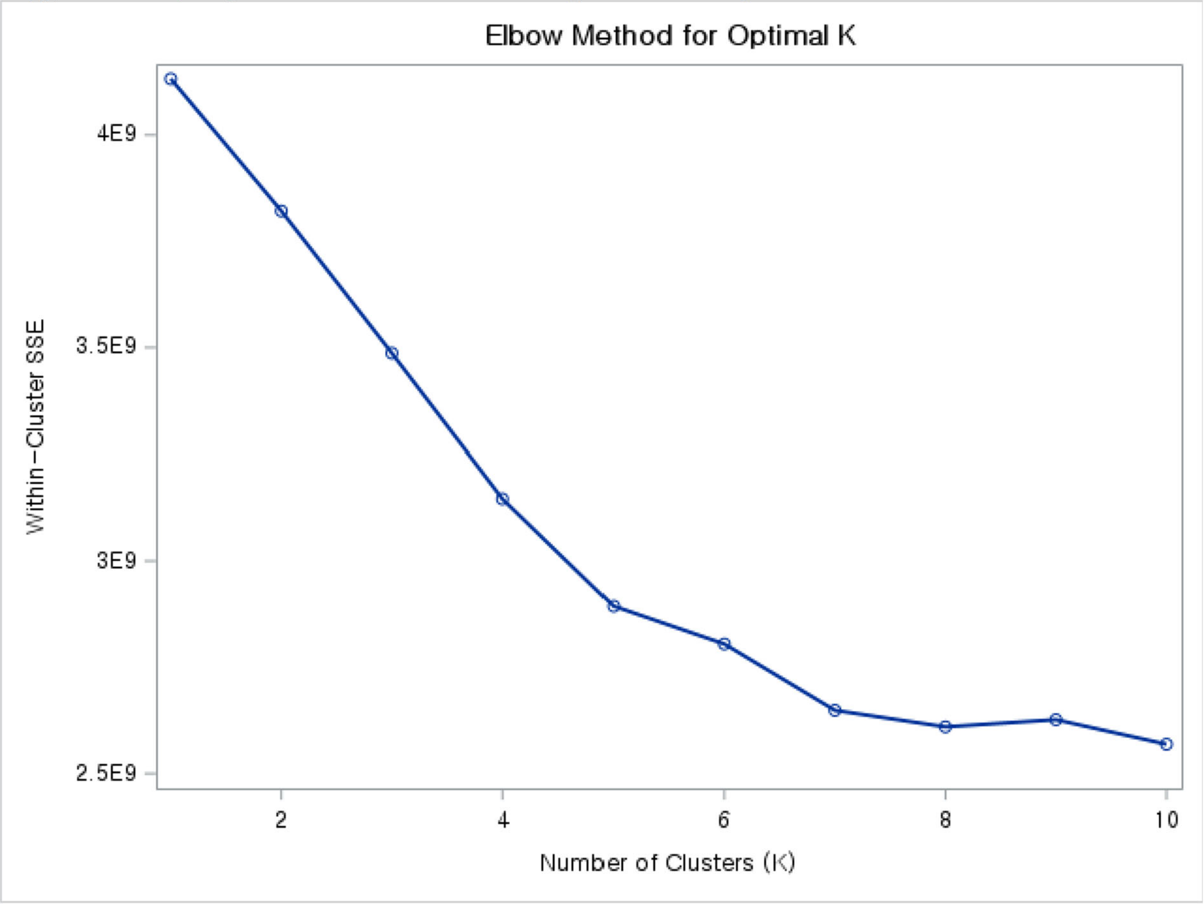

Description: To justify the selection of meal-timing chronotypes, we evaluated the within-cluster SSE for *K* values ranging from 1 to 10. The resulting Elbow plot illustrates a distinct 'elbow' at *K*=3, where the marginal gain in cluster compactness (measured by SSE reduction) begins to diminish. Beyond *K*=5, the curve levels off significantly, suggesting that increasing the number of clusters further would result in over-fitting without providing additional meaningful patterns. Therefore, *K*=3 was identified as the most stable and statistically sound number of clusters for our analysis.

Supplementary Table S1. Comparison of nutrient intake and Korean Healthy Eating Index scores by meal-timing chronotype among participants.

		**Total (n = 3465)**			
		**Morning Preference (n = 1410)**	**Intermediate Preference (n = 1173)**	**Evening Preference (n = 882)**	***p***
**HEI** [(1)]					
Total HEI		64.4 ± 0.4 ^{c}	60.9 ± 0.4 ^{b}	59.8 ± 0.5 ^{a}	**<0.001**
Adequacy items	Breakfast	7.0 ± 0.1 ^{c}	6.0 ± 0.1 ^{b}	5.3 ± 0.2 ^{a}	**<0.001**
	Whole grain	1.9 ± 0.1 ^{c}	1.6 ± 0.1 ^{b}	1.3 ± 0.1 ^{a}	**<0.001**
	Fruits (any type)	2.0 ± 0.1 ^{b}	1.9 ± 0.1 ^{b}	1.7 ± 0.1 ^{a}	**<0.001**
	Fruits (fresh only)	2.3 ± 0.1 ^{b}	2.1 ± 0.2 ^{b}	1.9 ± 0.1 ^{a}	**<0.001**
	Vegetables (any type)	3.6 ± 0.0 ^{b}	3.5 ± 0.1 ^{b}	3.3 ± 0.1 ^{a}	**<0.001**
	Vegetables (excluding pickled)	3.3 ± 0.0 ^{b}	3.1 ± 0.1 ^{a}	3.1 ± 0.1 ^{a}	**0.001**
	Protein sources	7.8 ± 0.1 ^{b}	7.4 ± 0.1 ^{a}	7.5 ± 0.1 ^{ab}	**0.005**
	Dairy products	3.8 ± 0.1^{b}	3.3 ± 0.5 ^{a}	2.8 ± 0.2 ^{a}	**<0.001**
	Total score	31.7 ± 0.3 ^{c}	28.8 ± 0.3 ^{b}	26.9 ± 0.4 ^{a}	**<0.001**
Moderation items	Saturated fat (%E)	7.0 ± 0.1 ^{b}	6.9 ± 0.2 ^{b}	6.1 ± 0.2 ^{a}	**<0.001**
	Sodium	6.3 ± 0.1	6.4 ± 0.1	6.5 ± 0.1	0.436
	Sugar (%E)	9.1 ± 0.1	8.9 ± 0.1	9.1 ± 0.1	0.059
	Total score	22.4 ± 0.2 ^{b}	22.1 ± 0.2 ^{a}	21.6 ± 0.3 ^{a}	**0.037**
Balance of energy items	Carbohydrates (%E)	3.1 ± 0.1 ^{b}	3.0 ± 0.1 ^{b}	2.5 ± 0.1 ^{a}	**<0.001**
	Fat (%E)	3.9 ± 0.1 ^{b}	3.8 ± 0.1 ^{b}	3.2 ± 0.1 ^{a}	**<0.001**
	Energy adequacy	3.4 ± 0.1 ^{b}	3.2 ± 0.2 ^{b}	2.6 ± 0.1 ^{a}	**<0.001**
	Total score	10.4 ± 0.1 ^{b}	10.0 ± 0.2 ^{b}	8.3 ± 0.1 ^{a}	**<0.001**

Data are presented as mean ± standard error. *p*-values were obtained from survey-weighted linear regression, adjusted for age, sex, education level, occupation, household income, and physical activity. Calcium intake was additionally adjusted for total energy intake and the KHEI. Means with different superscript letters ($^{a, b, c}$) within a row indicate significant differences at $p < 0.05$ based on Tukey's post hoc test. [(1)] KHEI: Korean Healthy Eating Index. In this study, the total KHEI score was calculated out of a maximum of 100 points by summing 13 components: Adequacy (8 items, 55 points): breakfast (10), mixed grain intake (5), total fruit intake (5), fresh fruit intake (5), total vegetable intake (5), vegetable intake excluding kimchi and pickles (5), meat/fish/eggs/legumes intake (10), and milk and dairy products (10). Moderation (3 items, 30 points): Saturated fatty acid energy ratio (10), sodium intake (10), and sugar/beverage energy ratio (10); and balance (3 items, 15 points)—carbohydrate energy ratio (5) fat energy ratio (5), and energy adequacy (5).

Supplementary Table S2. Odds ratios (ORs) and 95% confidence intervals (CIs) for low calcium intake (T1) according to meal-timing chronotype among participants aged 30–49 years.

Meal-timing chronotype	Model 1	Model 2	Model 3
**Morning preference (ref)**	-	-	-
**Intermediate preference**	1.7(1.4–2.1)**	1.7(1.4–2.2)**	1.5(1.2–1.9)*
**Evening preference**	1.8(1.5–2.3)**	1.8(1.5–2.3)**	2.2(1.7–2.9)**

* $p < 0.01$, ** $p < 0.001$
p-values were obtained from survey-weighted multinomial logistic regression.
The morning preference group served as the reference group for the meal-timing chronotypes.
Model 1: adjusted for age and sex.
Model 2: Model 1 plus education level, occupation, and household income.
Model 3: Model 2 plus aerobic physical activity and total energy intake.
